# The role of Drosophila mismatch repair in suppressing recombination between diverged sequences

**DOI:** 10.1038/srep17601

**Published:** 2015-11-30

**Authors:** Anthony T. Do, Jeannine R. LaRocque

**Affiliations:** 1 Department of Human Science, Georgetown University Medical Center, Washington DC 20057.

## Abstract

DNA double-strand breaks (DSBs) must be accurately repaired to maintain genomic integrity. DSBs can be repaired by homologous recombination (HR), which uses an identical sequence as a template to restore the genetic information lost at the break. Suppression of recombination between diverged sequences is essential to the repair of DSBs without aberrant and potentially mutagenic recombination between non-identical sequences, such as Alu repeats in the human genome. The mismatch repair (MMR) machinery has been found to suppress recombination between diverged sequences in murine cells. To test if this phenomenon is conserved in whole organisms, two DSB repair systems were utilized in *Drosophila melanogaster*. The DR-*white* and DR-*white.mu* assays provide a method of measuring DSB repair outcomes between identical and diverged sequences respectively. *msh6*^*–/–*^ flies, deficient in MMR, were not capable of suppressing recombination between sequences with 1.4% divergence, and the average gene conversion tract length did not differ between *msh6*^*–/+*^ and *msh6*^*–/–*^flies. These findings suggest that MMR has an early role in suppressing recombination between diverged sequences that is conserved in Drosophila.

Maintaining genome integrity is essential for the survival of a cell and for accurate transmission of genetic information from generation to generation. One threat to the genome is DNA damage, which occurs from both exogenous and endogenous sources. A particularly deleterious type of DNA damage is the double-strand break (DSB), where both strands of the DNA double helix are broken. DSBs result from exogenous sources such as ionizing radiation, and endogenous cellular byproducts such as high-energy free radicals and single-strand breaks that are converted to DSBs during replication. DSBs can be repaired through homologous recombination (HR), non-homologous end-joining (NHEJ) or single-strand annealing (SSA)^1^ (Fig. 1). If not accurately repaired, DSBs can result in cell death, mutations, cancer, and premature aging^2^.

HR is a mechanism in which a homologous DNA sequence is used as a template to repair a DSB. In mitotic cells, the preferred template is an identical sister chromatid^3^. There are two pathways involved in repair via HR. Both pathways are initiated by 5′ to 3′ resection (Fig. 1) of the recipient sequence, followed by Rad51-dependent strand invasion^4^,^5^. Strand invasion into the donor sequence results in formation of a D-loop, after which the mechanisms for the two HR pathways differ. In the DSB repair (DSBR) model, D-loop formation is followed by the formation of a double Holliday junction (dHJ) (Szostak, 1983). Resolution of the dHJ can result in a crossover or noncrossover product (Fig. 1A). The alternative pathway, synthesis-dependent strand annealing (SDSA), involves repair synthesis, dissociation of the newly synthesized strand, and ligation to the other DNA end resulting in a noncrossover product (Fig. 1B). DSBR can result in a crossover product, which is essential in meiosis, while SDSA is the preferred pathway for mitotically dividing cells.

HR using an identical sequence is highly regulated, as recombination between diverged, or non-identical, sequences is suppressed in many organisms including yeast^6^, mammals^7^,^8^, and Drosophila^9^. Sequence divergence of up to 1.4% in murine embryonic stem cells was found to cause significant decreases in recombination^7^. This suppression is essential to prevent aberrant recombination between non-identical repetitive sequences, such as Alu repeats in humans^10^. It has been shown that failure to suppress recombination between diverged sequences can compromise genome stability, resulting in tumorigenesis and changes in chromosomal structure such as translocations^2^,^11^. Suppression of recombination between diverged sequences is due in part to the function of the mismatch repair (MMR) machinery^6^,^8^,^12^,^13^, which recognize mismatches formed in heteroduplex DNA (hDNA) where one strand is from the donor sequence used as a template for repair and the other from the sequence containing the DSB. Base-base mismatches and insertion/deletion loops in hDNA are targeted by MMR for repair. HR intermediates in both DSBR and SDSA contain hDNA (Fig. 1, boxes).

MMR is initiated when an Msh2/Msh6 heterodimer^14^ recognizes and binds to DNA mismatches in an ATP-dependent manner, and an MLH1/PMS2 heterodimer facilitates this process and subsequent steps^15^. Exo1 nuclease is recruited to the lesion, digests the lesion, and the sequence is filled-in by a translesional or replicative DNA polymerase^16^,^17^. The key proteins in both HR and MMR are highly conserved, with multiple homologs from prokaryotic organisms having been identified in eukaryotic organisms^18^. MMR proteins have been found to suppress recombination between diverged sequences in yeast^6^,^13^, *E. coli*^12^, and murine cells^8^. The mechanism by which MMR proteins suppress recombination has yet to be elucidated, but it is thought that MMR can detect base-base mismatches in hDNA found in recombination intermediates. In the event of high sequence divergence, MMR can then recruit downstream proteins that then abort the HR process. In the absence of MMR, no downstream proteins are recruited to abort recombination, despite any divergence in sequence^13^,^19^.

It is unknown if the role of MMR in suppressing recombination between diverged sequences is conserved in multicellular systems. This question was addressed for the first time in the context of a multicellular organism using the genetically tractable organism *Drosophila melanogaster*. The DR-*white* and DR-*white.mu* assays are capable of detecting the repair pathway of an induced simple DSB (Fig. 2), including HR, NHEJ and SSA^9^. In the DR-*white.mu* assay, the donor sequence contains 28 silent polymorphisms, resulting in an increase of sequence divergence to 1.4%. To determine whether MMR plays a role in the suppression of recombination between diverged sequences in Drosophila, the DR-*white* and DR-*white.mu* assays were used with *msh6* mutant flies^20^. We found that while HR repair between identical sequences, NHEJ, and SSA were unaffected by MMR deficiency, *msh6*^*–/–*^ flies were not capable of suppressing recombination between diverged sequences compared to *msh6*^*–/+*^ heterozygote controls. Futhermore, gene conversion tract (GCT) lengths between *msh6*^*–/–*^ and *msh6*^*–/+*^ flies were found to be similar. These findings suggest that MMR suppresses recombination between diverged sequences in Drosophila and the similar GCT lengths suggest that MMR components act on the repair intermediate prior to repair synthesis.

## Results

### MMR machinery is required for suppression of recombination in Drosophila between diverged sequences

**Figure 2:**
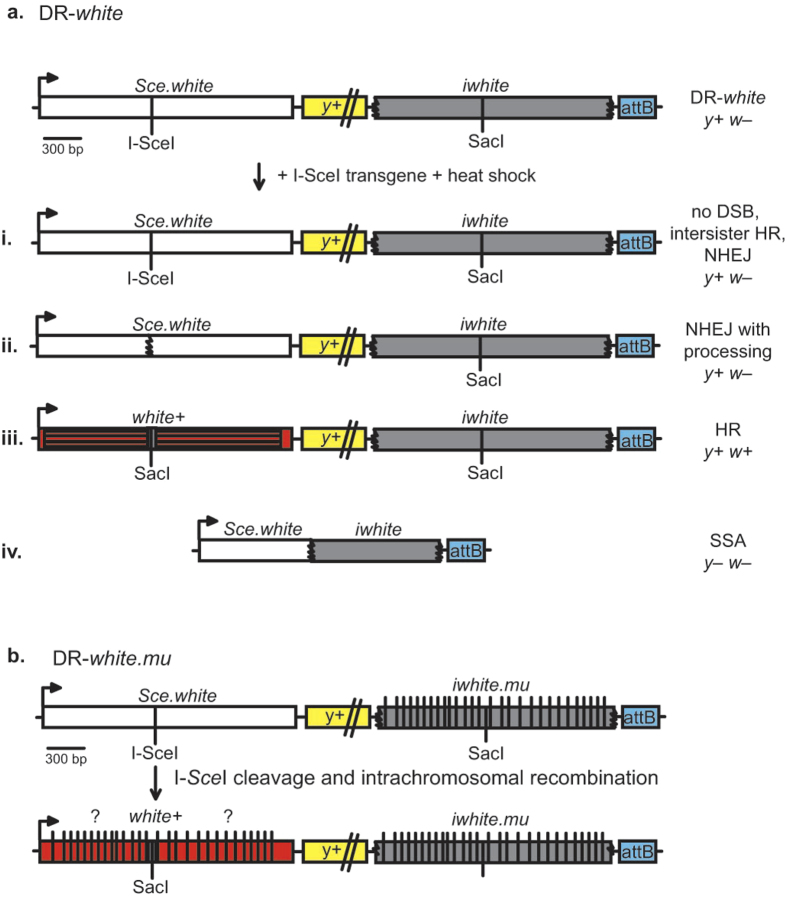
DR-*white* and DR-*white.mu* DSB Repair Assays. (**a**) The DR-*white* assay contains two nonfunctional direct repeats of the *white* gene. The first repeat, *Sce.white*, is nonfunctional due to the insertion of an I-SceI recognition sequence into the wild-type SacI recognition sequence of *white* cDNA resulting in a defective *white* gene. The second repeat, *iwhite*, is nonfunctional due to 5′ and 3′ truncations. DR-*white* is targeted using the attB sequence and integration is confirmed using *yellow* (*y* + ) transgene expression. DR-*white* flies are crossed with flies containing the heat-shock inducible I-SceI transgene, followed by heat shock, which results in DSB formation at the I-SceI recognition sequence. One of four repair products will result. White-eyed progeny (*y*^+^*w*^*–*^) suggest (**i**) no DSB or repair by (**ii**) NHEJ with processing, resulting in loss of the I-SceI recognition sequence. These two outcomes can be distinquished through molecular analysis. (**iii**) Repair by HR results in restoration of the wild-type SacI site and a red-eyed fly (*y*^*+*^*w*^*+*^). (**iv**) Yellow-bodied white-eyed (*y*^*–*^*w*^*–*^) progeny indicates repair by SSA. (**b**) The DR-*white.mu* assay includes the incorporation of 28 silent polymorphisms on the *iwhite* sequence, resulting in a sequence divergence of 1.4% between the two direct repeats. The silent polymorphisms allow recombination between diverged sequences to be studied as well as determining the length and direction of gene conversion tracts. Conversion of each of the polymorphisms varies from one repair product to the next (indicated by “?”), and can be determined by molecular analyses.

To determine if MMR proteins suppress recombination between diverged sequences in *Drosophila melanogaster*, the DR-*white* and DR-*white.mu* assays^9^ were used in MMR-deficient (*msh6*^–/–^) flies. Briefly, the DR-*white* and DR-*white.mu* reporter assays repair DSBs induced by cleavage of a specific recognition sequence by the rare-cutting homing meganuclease I-SceI (Fig. 2). After heat shock, the heat-inducible I-SceI enzyme cleaves at the I-SceI recognition sequence of *Sce.white* and four possible outcomes occur (Fig. 2a). *y* + *w–* progeny result from: no DSB formation, repair by intersister HR, or repair by NHEJ (Fig. 2ai,ii). NHEJ with processing can be detected from this group by molecular analysis at the I-SceI site; loss of the I-SceI recognition sequence suggests repair by NHEJ with processing. Accurate repair by intrachromosomal noncrossover HR results in restoration of the wild-type SacI site and *w* + expression (Fig. 2Aiii). y– w– progeny results from loss of the *y* + coding sequence by extensive end resection followed by SSA of the direct repeat sequence (Fig. 2aiv). DR-*white.mu* is identical to DR-*white*, but contains 28 silent polymorphisms distributed along the length of the *iwhite* donor sequence. As with the DR-*white* DSB repair assay, repair by HR restores the wild-type *white* sequence (Fig. 2b), and NHEJ and SSA repair products can be detected as in Fig. 2a.

**Figure 3:**
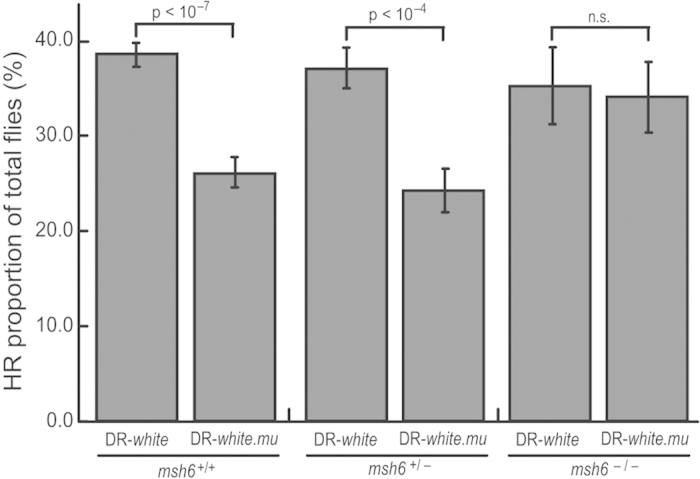
Msh6 suppresses recombination between diverged sequences. Recombination between homologous and diverged sequences in *Drosophila melanogaster* was determined using DR-*white* and DR-*white.mu*, respectively. Recombination between diverged sequences is suppressed in *msh6*^+/+^ flies (n = 117 and 67, respectively), as well as in the *msh6*^*–/+*^ heterozygote control (n = 48 and 46, respectively). However, suppression of recombination was not observed in *msh6*^*–/–*^ flies (n = 27 and 38, respectively). The average percentage of recombination with SEM is shown from 27–117 single male germlines representative of five independent experiments. Given P values were determined by unpaired t test. For total numbers, including SSA and NHEJ analysis, see Supplementary Tables 1 and 2. *msh6*^*+/+*^ data is from previous work^9^.

In a previous study, the DR-*white* assay determined that 39.4 ± 0.90% of progeny from *msh6*^*+/+*^ wild-type flies repaired DSBs via HR. With the incorporation of 28 silent polymorphisms increasing sequence divergence to 1.4% in the DR-*white.mu* assay, recombination was suppressed to 27.1% (±1.18%), a 31.5% suppression of recombination (P < 0.0000001 by unpaired t test; Fig. 3)^9^. Similar results were observed in *msh6*^*–/+*^ heterozygotes. Progeny from DR-*white*; *msh6*^*–/+*^ flies repaired DSBs via HR at a frequency of 36.6 ± 0.02% (703 of 1923), while progeny from DR-*white.mu*; *msh6*^*–/+*^ flies repaired via recombination at a frequency of 24.7 ± 0.02% (428 of 1736 flies; P < 0.0001, unpaired t test; Fig. 3), resulting in 34.3% suppression of recombination. However, this suppression of recombination between diverged sequences was not observed in *msh6*^*–/–*^ flies. Progeny from DR-*white*; *msh6*^*–/–*^ flies repaired DSBs via HR at a frequency of 40.7 ± 0.04% (261 of 645 flies), while progeny from DR-*white.mu*; *msh6*^*–/–*^ flies repaired via recombination at a frequency of 34.3 ± 0.03% (282 of 821 flies; P > 0.05, unpaired t test; Fig. 3, Supplementary Table S1).

SSA frequencies were also determined, with no significant difference between the DR-*white* or DR-*white.mu*, regardless of genotype (1.43 ± 0.50% SSA vs. 1.03 ± 0.33% SSA respectively for *msh6*^*–/+*^ and 1.23 ± 0.35% SSA and 1.05 ± 0.43% SSA respectively for *msh6*^*–/–*^ (P > 0.05, unpaired t test, Supplementary Table S1)). *y*^*+*^*w*^*–*^ flies were also analyzed for NHEJ with processing by PCR amplification of the *Sce.white* sequence followed by I-SceI restriction digest. There was no difference in NHEJ with processing frequency between identical and diverged sequences for both *msh6*^*–/+*^ (4.92% for DR-*white* vs. 5.45% for DR-*white.mu*; P > 0.05 by Fisher’s exact test, Supplementary Table S2) and *msh6*^*–/–*^ flies (8.57% for DR-*white* and 4.76% for DR-*white.mu;* P > 0.05 by Fisher’s exact test, Supplementary Table S2).

### Gene conversion tract analysis

**Figure 4:**
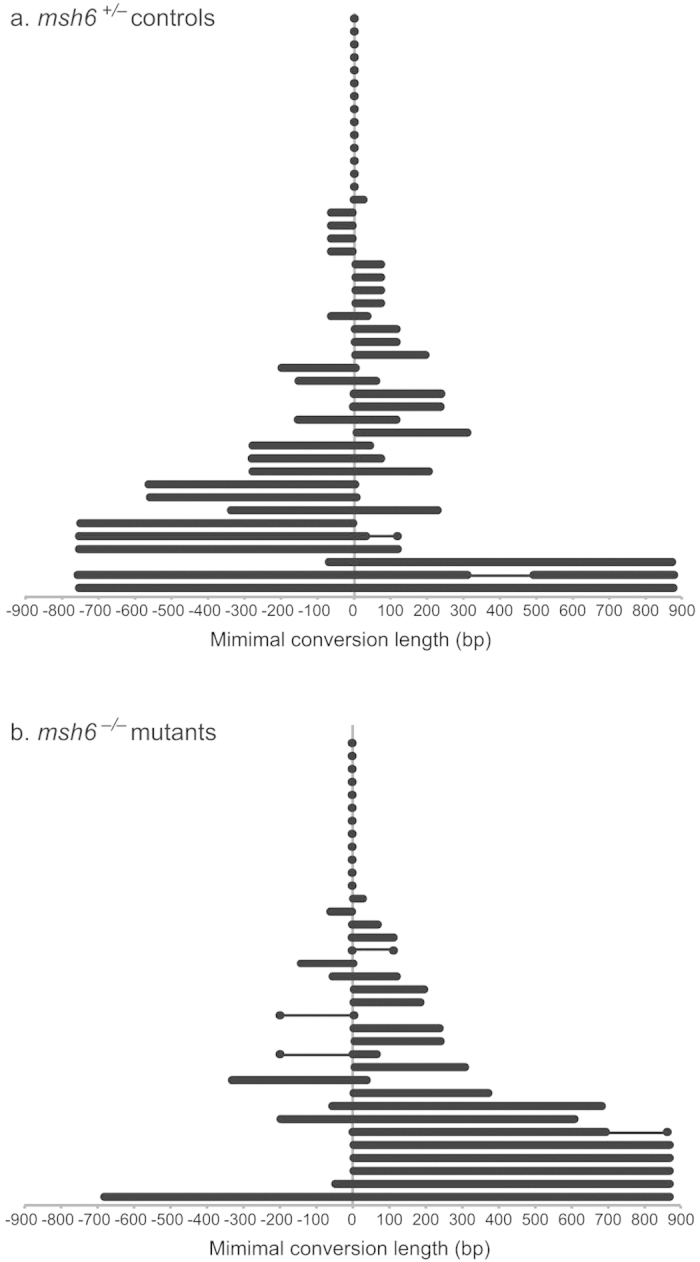
Gene conversion tract analysis. HR events using the DR-*white.mu* assay were isolated, and the GCT direction and length were determined. Briefly, *Sce.white* was amplified from *y*^*+*^*w*^*+*^(HR^+^) isolates and then sequenced for the conversion of polymorphisms to the *iwhite.mu* donor sequence. Minimal conversion lengths are displayed for (**a**) 45 *msh6*^*–/+*^ HR events and (**b**) 35 *msh6*^*–/–*^ HR events, including the last polymorphism converted. Discontinuous conversion is indicated by a thin line. The distance converted (bp) to the left and to the right of the SacI site (0) is given. Data are representative of four independent experiments.

To explore at which step in the HR pathway that MMR machinery functions to suppress recombination between diverged sequences, GCTs were analyzed in *y*^*+*^*w*^*+*^ (HR+) DR-*white.mu* flies. The average GCT of *msh6*^*–/+*^ and *msh6*^*–/–*^ flies were 264.0 ± 57.7 bp and 296.7 ± 65.3 bp respectively (Fig. 4; P > 0.05, by unpaired t test). Of all the *msh6*^*–/+*^ HR repair events (n = 45), 14 (31.1%) of the GCTs were limited to the SacI site, 19 (42.2%) were unidirectional, and 12 (26.7%) were bidirectional (Fig. 4). The unidirectional GCTs did not display a preference to either the left or right. Of all the *msh6*^*–/–*^HR repair events analyzed (n = 35), 12 (34.3%) were limited to the SacI site, 16 (45.7%) were unidirectional, and 7 (20%) were bidirectional (Fig. 4). *msh6*^*–/–*^HR repair events displayed directional gene conversion preference, with 13 (81.3%) of the unidirectional GCTs converting to the right side; however, this was not significant compared to the distribution of the direction of unidirectional tracts in *msh6*^*–/+*^ HR events (P > 0.05, Fisher’s exact test). Discontinuous GCTs were also observed, where conversion along the entire length of the gene conversion tract did not occur. Two discontinuous tracts were observed in *msh6*^*–/+*^ flies and 4 were observed in *msh6*^*–/–*^ flies (P > 0.05, Fisher’s exact test; Fig. 4).

## Discussion

Findings from previous studies suggest that the role of MMR proteins in suppressing recombination between diverged sequences is conserved across yeast^6^,^13^, *E. coli*^12^, and murine cells^8^. This study provides evidence suggesting that suppression of recombination between diverged sequences is also conserved in *Drosophila melanogaster* and is observed in the context of a whole organism rather than single cells, as demonstrated here. While not as large as the percentage estimated in the human genome^21^, the *Drosophila melanogaster* genome comprises between 8 to 12% repetitive sequences^22^,^23^,^24^, eliciting the need for stringent regulation and suppression of aberrant and potential mutagenic recombination between diverged sequences.

With a suppression of recombination between diverged sequences in *msh6*^*–/+*^ flies, the proportion of NHEJ or SSA repair is expected to increase. However, repair through these pathways did not differ significantly between identical or diverged sequences. One explanation is that in the DR-*white* and DR-*white.mu* assays, intersister HR may occur when the *Sce.white* sequence of the broken strand alternatively uses the *Sce.white* sequence of a sister chromatid as a donor sequence template for repair. *msh6*^*–/+*^ flies may be capable of detecting mismatches in hDNA and prevent repair synthesis from occurring using the downstream diverged *iwhite.mu* sequence. The broken sequence can then proceed to repair itself through intersister HR if a sister chromatid is available. However, this cannot be directly tested since the DR-*white* and DR-*white.mu* assays are unable to specifically detect intersister HR, as these repair products are phenotypically and molecularly identical to “no DSB” repair products and NHEJ without processing. A second explanation is that the cell may proceed through an apoptotic pathway if a sister chromatid is not available. However, a limitation of this system is that an unrepaired DSB that results in apoptosis cannot be observed/characterized. A third possible explanation is that mismatch repair deficiency may impact cutting frequency; however, this is unlikely since the distribution of repair events in the DR-*white* reporter are not different between *msh6*^*–/+*^ and *msh6*^*–/–*^ flies, suggesting similar DSB induction between genotypes.

*msh6*^*–/+*^ and *msh6*^*–/–*^ flies both displayed similar GCT lengths, suggesting that once repair synthesis is initiated, the MMR-deficient organism is capable of repairing DSBs through HR with similar repair synthesis. These findings implicate MMR in suppressing recombination between diverged sequences in earlier steps of HR (i.e., before repair synthesis). It is possible that MMR proteins are recruited during strand invasion and bind to mismatches in hDNA, consequently serving as a scaffold to recruit other proteins that may directly suppress recombination between diverged sequences. Such proteins could include a helicase such as BLM that aborts D-loop formation, and consequently aborts the aberrant repair product^13^,^19^. This study lays the foundation for future work in *Drosophila melanogaster* utilizing a unique genetic tool to gain a better understanding of this complex and highly regulated process in maintaining genome integrity in multicellular systems.

## Methods

### Drosophila Stocks and Genetics

Drosophila were maintained on standard Nutri-fly Bloomington Formulation medium (Genesee Scientific; San Diego, CA) at 25 °C. Standard genetic crosses were used to create two stocks containing a DR-*white; msh6*^*68*^ mutant allele and DR-*white.mu*; *msh6*^*68*^ mutant allele (Radford, 2007), both in a *y w* background.

### Drosophila DSB Repair Assay

To induce DSBs, females containing DR-*white* or DR-*white.mu* with a mutant *msh6*^*68*^ allele were crossed to males containing the heat-inducible I-SceI transgene^25^ with the mutant *msh6*^10^ allele^20^. After 3 days, flies were removed and 0 to 3 day old embryos were heat shocked in a 38 °C water bath for 1 h. Single *msh6*^*–/+*^ or *msh6*^*–/–*^ males from this cross containing DR-*white* or DR-*white.mu* and the heat inducible I-SceI transgene were crossed to 4–5 *y w* virgin females. The progeny from these single male crosses represent single repair events from each male germline. Phenotypes of progeny from individual male germlines were scored. Brown-bodied and white-eyed (*y*^*+*^*w*^*–*^) progeny indicate either no DSB occurred (Fig. 2ai) or repair through NHEJ (Fig. 2aii). Red-eyed progeny (*y*^*+*^*w*^*+*^) indicates repair through HR (Fig. 2aiii). Lastly, yellow-bodied and white-eyed (*y*^*–*^ *w*^*–*^) progeny indicate repair through SSA (Fig. 2aiv).

### Molecular Analysis

Genomic DNA was isolated from individual flies using 50 μL Squishing Buffer (10 mM Tris-Cl, 25 mM NaCl, 1 mM EDTA) and Proteinase K (0.2 mg/mL). Samples were incubated at 37 °C for 30 m, followed by inactivation of Proteinase K by heating to 95 °C for 2 m^22^. *Sce.white* was PCR amplified using *Sce.white* specific primers (forward, DR-*white*1.3, 5′ GTTTTGGGTGGGTAAGCAGG; reverse, DR-*white*1a 5′ AGACCCACGTAGTCCAGC) and SapphireAmp Fast PCR Master Mix (Clonetech; Mountain View, CA, USA). For NHEJ junction analyses, *Sce.white* PCR products were directly digested with I-SceI restriction enzyme (New England Biolabs; Ipswitch, MA). The PCR product was visualized on a 1% agarose gel.

### Gene Conversion Tracts

*Sce.white* in DR-*white.mu* HR events was amplified with DR-*white1.3* and DR-*white* 1a as described above, and *Sce.white* PCR products were directly sequenced (Genewiz) with primers DR-*white* 2 (5′ ATGCAGGCCAGGTGCGCCTATG); DR-*white* 2a (5′ TGGCAACCATCGTTGTCTG); DR-*white* 1.3 (5′ GTTTTGGGTGGGTAAGCAGG0; and *DR-white* 1a (5′ AGACCCACGTAGTCCAGC). for incorporations of any of the 28 silent polymorphisms. Sequences were analyzed using Chromas (Technelysium; South Brisbane, Australia).

## Additional Information

**How to cite this article**: Do, A. T. and LaRocque, J. R. The role of Drosophila mismatch repair in suppressing recombination between diverged sequences. *Sci. Rep.* **5**, 17601; doi: 10.1038/srep17601 (2015).

## Supplementary Material

Supplementary Information

## Figures and Tables

**Figure 1:**
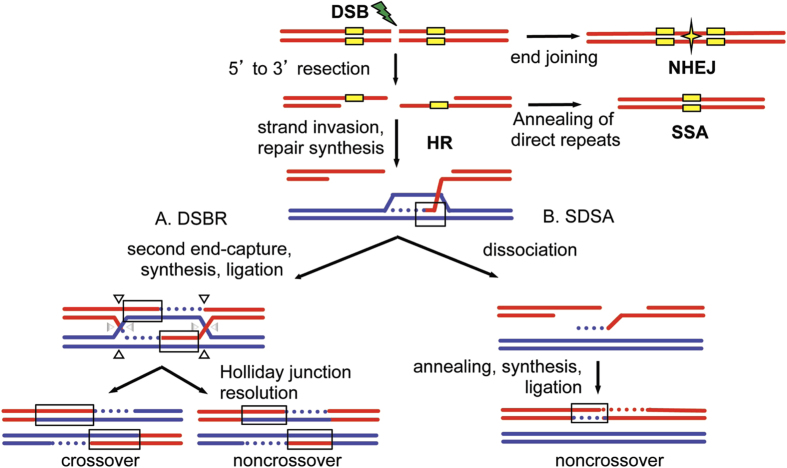
Models of DSB repair. DSBs can be repaired by homologous recombination (HR), single-strand annealing (SSA) or non-homologous end joining (NHEJ). In NHEJ, processed ends are joined by ligation (star). HR repair is initiated by 5′ to 3′ resection at the DSB. If the DSB occurs between direct repeats (yellow boxes), extensive resection followed by annealing of the direct repeats results in SSA, resulting in loss of the intervening sequence. Alternatively, the resected 3′ overhang invades the homologous template (blue) to initiate repair synthesis (blue dotted line). The invaded strand may result in heteroduplex DNA between the red and blue sequences (hDNA, black box). DNA repair synthesis is then initiated. (**A**) In the DSBR model, the second strand of the DSB is captured, followed by repair synthesis, and then the newly synthesized strands are ligated to form a double Holliday junction (dHJ). Depending on how the dHJ is cleaved (arrow heads), resolution can result in a crossover or a noncrossover. (**B**) In SDSA, the newly synthesized strand dissociates, anneals to the other end, the gap is filled in, and nicks ligated to result in a noncrossover product. The newly synthesized strands in both DSBR and SDSA also form hDNA. hDNA in these products can be repaired by mismatch repair, resulting in gene conversion (not shown). Direct repeats are shown only for SSA for simplicity.
