# Supplementary material for: The role of Drosophila mismatch repair in suppressing recombination between diverged sequences
Source: Sci Rep. 2015 Nov 30;5:17601. doi: 10.1038/srep17601 (PMC4663472; doi:10.1038/srep17601)
Supplement: Supplementary Information [file srep17601-s1.pdf]

Supplementary Information

The role of Drosophila mismatch repair in suppressing recombination between diverged sequences

Anthony T. Do and Jeannine R. LaRocque

Supplementary Table S1– Total Flies Scored for Each DSB Repair Phenotype

Genotype	DSB repair assay	Experiment number	No. Progeny (No. of individual germlines)	Total number of each phenotype (% of total)		
				y+ w– NHEJ, intersister HR, no DSB	y+ w+ intra-chromosomal HR	y–w– SSA
msh6$^{-/+}$	DR-*white*	1	284 (11)	184 (64.8)	94 (33.1)	6 (2.11)
		2	282 (13)	168 (59.6)	106 (37.6)	8 (2.84)
		3	73 (3)	41 (56.2)	32 (43.8)	0 (0.00)
		4	127 (8)	69 (54.3)	57 (44.9)	1 (0.787)
		5	1157 (13)	729 (63.0)	414 (35.8)	14 (1.21)
		**Total**	**1923 (48)**	**1191 (61.9)**	**703 (36.6)**	**29 (1.51)**
		**Mean % (±S.E.M.)**		**59.6 (±1.98)**	**39.0 (±2.29)**	**1.43 (±0.496)**
	DR-*white.mu*	1	268 (12)	206 (76.9)	58 (21.6)	4 (1.49)
		2	153 (10)	112 (73.2)	38 (24.8)	3 (1.96)
		3	106 (4)	77 (72.6)	28 (26.4)	1 (0.943)
		4	117 (7)	84 (71.8)	33 (28.2)	0 (0.00)
		5	1092 (13)	813 (74.5)	271 (24.8)	8 (0.733)
		**Total**	**1736 (46)**	**1292 (74.4)**	**428 (24.7)**	**16 (0.922)**
		**Mean % (±S.E.M.)**		**73.8 (±0.892)**	**25.2 (±1.09)**	**1.03 (±0.334)**
msh6$^{-/-}$	DR-*white*	1	113 (4)	68 (60.2)	44 (38.9)	1 (0.885)
		2	275 (7)	137 (49.8)	136 (49.5)	2 (0.727)
		3	48 (3)	35 (72.9)	12 (25.0)	1 (2.08)
		4	93 (8)	66 (71.0)	27 (29.0)	0 (0.00)
		5	116 (5)	69 (61.1)	42 (37.2)	5 (1.77)
		**Total**	**645 (27)**	**375 (58.4)**	**261 (40.7)**	**9 (1.40)**
		**Mean % (±S.E.M.)**		**63.0 (±4.17)**	**27.6 (±2.62)**	**1.23 (±0.353)**
	DR-*white.mu*	1	361 (16)	254 (70.4)	101 (28.0)	6 (1.66)
		2	273 (9)	145 (53.1)	123 (45.1)	5 (1.83)
		3	11 (2)	11 (100)	0 (0.00	0 (0.00)
		4	63 (6)	42 (66.7)	21 (33.3)	0 (0.00)
		5	113 (5)	74 (65.5)	37 (32.7)	2 (1.77)
		**Total**	**821 (38)**	**526 (63.8)**	**282 (34.3)**	**13 (1.55)**
		**Mean % (±S.E.M.)**		**71.1 (±7.78)**	**27.8 (±7.50)**	**1.05 (±0.430)**

Supplementary Table S2 – Frequency of Nonhomologous End-Joining Events

DSB repair assay	Genotype	Experiment number	No. y+w– isolates analyzed	No. NHEJ with processing (%)
DR-*white*	*msh6*$^{-/+}$	1	3	0 (0)
		2	13	1 (7.69)
		3	11	0 (0)
		4	9	0 (0)
		5	25	2 (8.00)
		**Total**	**61**	3 (4.92)
	msh6$^{-/-}$	1	0	0 (0)
		2	7	0 (0)
		3	6	2 (33.3)
		4	12	1 (8.33)
		5	10	0 (0)
		**Total**	**35**	3 (8.57)
DR-*white.mu*	*msh6*$^{-/+}$	1	2	1 (50)
		2	11	0 (0)
		3	5	0 (0)
		4	11	0 (0)
		5	26	2 (7.69)
		**Total**	**55**	3 (5.45)
	msh6$^{-/-}$	1	14	1 (7.14)
		2	10	1 (10)
		3	1	0 (0)
		4	7	0 (0)
		5	10	0 (0)
		**Total**	**42**	2 (4.76)
